# On the Accuracy of *In Vivo* Ethanol and Acetaldehyde Monitoring, a Key Tile in *the Puzzle of Acetaldehyde as a Neuroactive Agent*

**DOI:** 10.3389/fnbeh.2017.00097

**Published:** 2017-05-30

**Authors:** Paolo Enrico, Marco Diana

**Affiliations:** ^1^Department of Biomedical Sciences, University of SassariSassari, Italy; ^2^‘G. Minardi’ Cognitive Neuroscience Laboratory, Department of Chemistry and Pharmacy, University of SassariSassari, Italy

**Keywords:** ethanol, acetaldehyde, magnetic resonance spectroscopy, microdialysis, biosensors

## Abstract

Over the last 20 years researchers have explored the postulated role of acetaldehyde (ACD) as a mediator of some of the actions of ethanol (EtOH) in the central nervous system (CNS). However, efforts have been hampered mainly by the difficulty of directly measuring *in vivo* EtOH and ACD levels in the CNS and thus, our knowledge is based on indirect evidences. Although technically challenging, the development of reliable methods for *in vivo* measurement of ACD and EtOH is of paramount importance to solve the “*puzzle of acetaldehyde as a neuroactive agent.*” In this short review we discuss the recent advances on brain EtOH pharmacokinetic and state-of-the-art available techniques that could be used for *in vivo* detect EtOH and ACD both non-invasively (magnetic resonance spectroscopy), and invasively (microdialysis and biosensors). Among the different *in vivo* sampling techniques described, particular emphasis is paid to the field of enzyme-based amperometric biosensors. Biosensors have gained much attention in recent years for their ability to online monitor biological signals *in vivo*, and several micro- and nano-structured devices have been successfully used for *in vivo* studies. Owing to their high temporal and spatial resolution, biosensors could provide the adequate technology for studying *in vivo* EtOH pharmacokinetic.

## Introduction

Acetaldehyde (ACD) is a naturally occurring compound, found in several fruits and vegetables as well as in tobacco smoke and fermented alcoholic beverages (Cao et al., 2007).

In the last decades many attempts have been made to quantify brain EtOH and ACD, in order to correlate their concentrations with behavior (Correa et al., 2012; Israel et al., 2015). So far this line of research has yielded conflicting results, mostly due to discrepancy and controversy with quantitative measures of brain EtOH and ACD.

In this brief review we offer an overview of the recent advances on brain EtOH pharmacokinetic and discuss the state-of-the-art of available techniques for *in vivo* EtOH and ACD study.

## EtOH Metabolism in the Brain

Since EtOH readily enters the brain, *in situ* synthesis has been long postulated as a plausible source of brain ACD (Cohen et al., 1980). It is now demonstrated that the brain tissue contains all of the main EtOH metabolizing enzymes: alcohol dehydrogenase (ADH), cytochrome P450 2E1 (CYP2E1), and catalase; however, their relative role in metabolizing EtOH into ACD is still debated.

Alcohol dehydrogenase is a zinc-containing enzyme localized in the cytosol, it has broad substrate specificity (many primary or secondary alcohols) and is found in highest amount in the liver. However, since ADH is not uniformly expressed in the brain tissue, its real contribution to local ACD levels in discrete brain areas could have been underestimated and may deserve more detailed evaluation (Bühler et al., 1983; Kerr et al., 1989; Mori et al., 2000).

Cytochrome P450s are a family of heme enzymes mainly located in the endoplasmic reticulum and in mitochondria. CYP2E1 is the P450 family with the highest activity for oxidizing EtOH to ACD, and is widely expressed in the human and rodent brain (Tindberg and Ingelman-Sundberg, 1996; Sánchez-Catalán et al., 2008; Ferguson and Tyndale, 2011). CYP2E1 has been shown to metabolize EtOH in both neurons and astrocytes at a rate of 0.00051 μmol/min/g, and CYP2E1 pharmacological inhibition significantly reduces ACD formation in rat brain homogenates incubated with EtOH (Hansson et al., 1990; Gill et al., 1992; Warner and Gustafsson, 1994; Zimatkin et al., 2006). Further, reduced ACD brain levels have been shown in transgenic KO CYP2E1 mice after incubation with EtOH, relative to their wild-type counterparts (Ziegler et al., 2006). CYP2E1 activity has been accounted for a 20% fraction of brain EtOH oxidation, and it may represents a major adaptive response to chronic EtOH consumption as shown in a recent *in vivo* study on EtOH-induced locomotion (Hansson et al., 1990; Heit et al., 2013; Ledesma et al., 2014). Further, *in vitro* evidences in KO CYP2E1, acatalasemic and double mutants (KO CYP2E1 and acatalasemic) mice, suggest that CYP2E1 function may be linked to catalase-mediated EtOH oxidation by increasing the availability of H_2_O_2_ (Halliwell, 2006; Zimatkin et al., 2006; Deng and Ra, 2008).

Catalase, a heme containing enzyme, is found in the peroxisomal fraction of the cell and can oxidize EtOH as shown in reaction 1.

(1) $$\begin{array}{c} {\mathrm{CH}}_{3}{\mathrm{CH}}_{2}\mathrm{OH}\;+\;H_{2}O_{2}\;\to \;{\mathrm{CH}}_{3}\mathrm{CHO}\;+\;{2H}_{2}O \end{array}$$

Recent results also show that 3-amino-1,2,4-triazole (3AT) administration impair the acquisition of operant EtOH self-administration in the rat (Peana et al., 2015). However, 3AT has been shown to cause a non-specific effects on behavior and therefore other procedures have been used to inhibit catalase-mediated ACD formation (Rotzinger et al., 1994; Tampier et al., 1995).

A valuable method for *in vivo* studying the involvement of catalase in brain ACD formation is based on the use of lentiviral vectors coding for an anticatalase shRNA (RNAi precursor), which allows for efficient (up to 75%) inhibition of catalase activity (Karahanian et al., 2011). This technique appears also of particular interest because by allowing localized inhibition of catalase activity, it may be used to precisely pinpoint those brain areas involved in the psychopharmacological effects of EtOH (Israel et al., 2015). In fact, it has been shown that administration of an anticalatase vector into the ventral tegmental area (area which plays a key role in the neurobiological basis of addiction, VTA), significantly reduced EtOH consumption and EtOH stimulated dopamine release in its projection fields (in particular the nucleus accumbens shell) (Karahanian et al., 2011; Israel et al., 2012; Quintanilla et al., 2012). Another study show that anticatalase vectors administration in the VTA can efficiently inhibit EtOH intake following deprivation (Tampier et al., 2013).

## *In Vivo* EtOH and ACD Detection: Non-Invasive Approaches

### Magnetic Resonance Spectroscopy

Magnetic resonance spectroscopy (MRS) is a non-invasive analytical technique used to provide a measure of *in vivo* brain biochemistry (Soares and Law, 2009; Strózik-Kotlorz, 2014; Buonocore and Maddock, 2015). *In vivo* MRS can be performed with common clinical magnetic resonance imaging equipments and since EtOH methyl protons can be detected (Sammi et al., 2000), MRS has been largely used to measure *in vivo* brain EtOH levels in both humans and laboratory animals (Hanstock et al., 1990; Kaufman et al., 1994; Rooney et al., 2000; Zahr et al., 2010). However, magnetic transfer evidences have clearly shown the *in vivo* presence of a free, observable EtOH pool and a membrane-associated EtOH pool that escapes direct detection (Fein and Meyerhoff, 2000; Nagel and Kroenke, 2008). Therefore, since a (possibly significant) fraction of brain EtOH content cannot be measured by MRS, this technique must be considered only for qualitative measurements.

Ethanol oxidative metabolism has been studied with MRS, after ^13^C-labeled EtOH administration (Xiang and Shen, 2011; Wang et al., 2013a,b). The results show that ^13^C nuclei from ^13^C-labeled EtOH are incorporated into multiple metabolites including glutamate, glutamine, and aspartate, but no significant conversion of EtOH into ACD in the brain could be evidenced.

Despite the low sensitivity and temporal resolution, MRS still provides an opportunity for *in vivo* qualitative study of the effects of EtOH in the brain (Nagel and Kroenke, 2008; Niciu and Mason, 2014). MRS is fundamental for human studies allowing the dynamic evaluation of EtOH effects, and providing an important framework for comparing experimental results in humans and animal models (Cifuentes Castro et al., 2014). Further, since magnetic resonance images can be obtained concurrently with spectroscopic data, MRS also provides valuable structural informations (Alger, 2010; Befroy and Shulman, 2011).

## *In Vivo* EtOH and ACD Detection: Invasive Approaches

### Microdialysis

Microdialysis is *de facto* the gold-standard *in vivo* sampling technique for the central nervous system (CNS), allowing the analysis of several molecules in cerebro spinal fluid (CSF) based on their diffusion across a semi-permeable membrane (Chefer et al., 2009; Kennedy, 2013). Despite its popularity, microdialysis is not free of limitations (Westerink, 1995), some of which are particularly relevant for EtOH and ACD *in vivo* measurement. In particular, due to low probe recovery, the concentrations of substances in the dialysate only partially reflect true tissue concentrations and thus, analytes present at very low concentrations are difficult to detect. This issue could be also worsened by the fact that some compounds may be adsorbed by the dialysis membrane further decreasing probe recovery (Buttler et al., 1996). It is well-known that microdialysis has poor time resolution and therefore is not suitable for studying events that change in short time intervals. Another problem is the effect of tissue damage secondary to probe implantation; although microdialysis probes have been miniaturized in time, alterations in tissue metabolism cannot be neglected (Borland et al., 2005; Carson et al., 2015).

Despite all technical shortcomings, the advantages of using microdialysis for *in vivo* monitoring of brain neurochemistry are clear. Microdialysis is a well-known and widely reproduced technique, sampling can be performed on freely moving subjects, and long-term studies can be carried out with minimal influence on the brain tissue physiology (Westerink, 1995; Chefer et al., 2009; Kennedy, 2013). On these bases, several authors used brain microdialysis to study EtOH and ACD *in vivo* (Yoshimoto and Komura, 1993; Jamal et al., 2003, 2007, 2015). However, this approach yielded only limited results, mostly due to the various technical and analytical issues which specifically impair the usefulness of microdialysis for EtOH and (especially) ACD *in vivo* monitoring.

#### High Performance Liquid Chromatography (HPLC)

High performance liquid chromatography (HPLC)-based analytical methods are largely employed in microdialysis studies (Cheng et al., 2009; Guihen and O’Connor, 2009). With regard to EtOH and ACD determination HPLC-based methods appear particularly suitable, since samples are not heated during analysis and thus heat sensitive or volatile compounds (such as EtOH and ACD) can be efficiently separated. Several protocols for EtOH analysis with HPLC have been developed using flame ionization detection (Yarita et al., 2002), ultraviolet detection after conversion to acetaldehyde-phenylhydrazone (Pellegrino et al., 1999), indirect photometric detection (Takeuchi et al., 1988), and enzymatic assay (Kristoffersen and Smith-Kielland, 2005; Peris et al., 2006). An optimized HPLC-based protocol for ACD determination in biological samples after derivatization with dinitrophenylhydrazine (Vogel et al., 2000) and diode array detector is also available (Guan et al., 2011).

#### Gas Chromatography (GC)

Gas chromatography (GC) is an efficient analytical technique for separating volatile species in complex samples, and several GC-based protocols have been developed for the detection of EtOH and ACD in biological matrices.

Several detector types can be used in conjunction with GC for EtOH and ACD detection; the most efficient protocols available have been developed mainly using mass spectrometry (GC–MS) (Heit et al., 2016) or flame ionization (GC-FID) (Chun et al., 2016), alone or in combination (Tiscione et al., 2011).

Gas chromatography with headspace extraction and mass spectrometry or flame ionization detection is the most reliable and sensitive technique available for EtOH and ACD detection in microdialysates (Xiao et al., 2014; Heit et al., 2016). Indeed, owing to their robustness and reliability, GC-based EtOH analysis are the gold standard technique for blood alcohol concentration measurement in forensic and toxicological laboratories (Cordell et al., 2013; Xiao et al., 2014; Goullé and Guerbet, 2015).

#### Fluorimetry

A new fluorimetry-based analytical method for EtOH and ACD has been recently published (Zachut et al., 2016). Although not specifically developed for EtOH and ACD detection in brain dialysates, this technique appears quite compatible with microdialysis; in particular: small sample volume, no sample pre-processing, simple methodology, relatively inexpensive laboratory equipment. Further, the limits of detection of the technique are reported to be comparable with the performance of GC methods.

### Biosensors

A biosensor can be defined as “a self-contained analytical device that combines a biological component with a physicochemical device for the detection of an analyte of biological importance” (Hasan et al., 2014). Biosensors typically consist of two key components: (1) a biological recognition element to detect the analyte; (2) a transducer to convert the biological response into a convenient output signal.

Among the different devices available, amperometric enzyme-based biosensors (AEBs) are increasingly employed in *in vivo* brain monitoring (Thévenot et al., 2001; Weltin et al., 2016). In fact, miniature (active surface – 1 mm, diameter – 150 μm) AEBs implantation induces reduced tissue damage, allows for real-time monitoring, with high sensitivity and specificity for analytes which cannot be studied with microdialysis (Timmerman and Westerink, 1997; Sirca et al., 2014). Another important feature of biosensors is the possibility of associating the implanted device to a telemetric system, allowing experiments in freely moving subjects (Olivo et al., 2011).

Amperometric enzyme-based biosensors are mainly based on enzymes that belong to two classes: oxidases and dehydrogenases; in their most common implementation the enzyme is linked on the transducer surface and the output signal is generated by measuring the electroactive by-products of enzymatic reaction.

In recent years several AEBs for EtOH detection have been developed, based on both alcohol oxidase (AOx) or dehydrogenase (ADH).

Alcohol oxidase catalyzes the oxidation of aliphatic, low molecular weight alcohols to their respective aldehydes using molecular oxygen (O_2_) as the electron acceptor and flavin-adenine dinucleotide (FAD) as cofactor (reactions 2 and 3).

(2) $$\begin{array}{c} {R-CH}_{2}\mathrm{OH}\;+\;AOx/FAD\;\to \;R-CHO\;+\;{AOx/FADH}_{2} \end{array}$$

(3) $$\begin{array}{c} {AOx/FADH}_{2}\;+\;O_{2}\;\to \;AOx/FAD\;+\;H_{2}O_{2} \end{array}$$

(4) $$\begin{array}{c} \;H_{2}O_{2}\;\to \;O_{2}\;+\;{2H}^{+}\;+\;{2e}^{-} \end{array}$$

The hydrogen peroxide produced by reaction 3 can be directly detected at the transducer surface of AOx-based AEBs (reaction 4). However, the high anodic potential needed to oxidize H_2_O_2_ poses a problem of Faradaic interference due to the presence of other compounds (such as ascorbic acid and uric acid) physiologically present in high concentrations in the CSF, which are also oxidized at the same potential (Belluzo et al., 2008). The use of a bi-enzyme AEB is a common way to circumvent this problem. In fact, by coupling a peroxidase [usually horseradish peroxidase (HRP)] to AOx it is possible to indirectly monitor EtOH-derived H_2_O_2_ at low working potentials reducing interfering signals (Vijayakumar et al., 1996; Azevedo et al., 2005).

(5) $$\begin{array}{c} \;H_{2}O_{2}\;+\;{2H}^{+}\;+\;{\mathrm{HRP}}^{-}\to {2H}_{2}O\;+\;{\mathrm{HRP}}^{+} \end{array}$$

The HRP^+^/HRP^-^ redox couple (reaction 5) is used as the sensing scheme at the transducer surface of AOx/HRP-based AEBs.

Alcohol dehydrogenase catalyzes the reversible oxidation of primary aliphatic and aromatic alcohols using nicotinamide-adenine dinucleotide (NAD) as cofactor (reaction 6).

(6) $$\begin{array}{c} \;{R-CH}_{2}\mathrm{OH}\;+\;{ADH/NAD}^{+}\;\to \;R-CHO\;+\;ADH/NADH\;+\;H^{+} \end{array}$$

(7) $$\begin{array}{c} \mathrm{NADH}\;\to \;{\mathrm{NAD}}^{+}\;+\;H^{+}\;+\;{2e}^{-}\; \end{array}$$

The most common way to monitor an ADH-catalyzed reaction is by using the NAD^+^/NADH redox couple (reaction 7) as the sensing scheme at the transducer surface of ADH-based AEBs (Lorenzo et al., 1998).

Acetaldehyde biosensors developed so far are based on ALDH, which catalyzes the oxidation of biogenic and xenobiotic aldehydes (including ACD) into acetate using NAD as cofactor (reaction 8).

(8) $$\begin{array}{c} R-CHO\;+\;{ALDH/NAD}^{+}\;\to \;R-COOH\;+\;ALDH/NADH\;+\;H^{+} \end{array}$$

(9) $$\begin{array}{c} \;\mathrm{NADH}\;\to \;\mathrm{NAD}\;+\;H^{+}\;+\;{2e}^{-} \end{array}$$

The ALDH-catalyzed reaction is monitored by using the NAD+/NADH redox couple (reaction 9) as the sensing scheme at the transducer surface of ALDH-based AEBs (Lorenzo et al., 1998).

Acetaldehyde biosensors have been mostly developed for toxicological and industrial purposes and therefore their biological applicative potential is much less characterized, when compared with EtOH AEBs. However the available evidence show that these devices can efficiently detect ACD in the μM range *in vitro*, with high time resolution and substrate specificity (Noguer and Marty, 1997; Noguer et al., 2001; Avramescu et al., 2002; Yao and Handa, 2003; Ghica et al., 2007).

Although the development of an adequate biosensor technology for *in vivo* EtOH and ACD detection is still in its infancy, the available evidence clearly show that this approach holds tremendous technological potential. In fact the prototypical properties of biosensors including high spatial and temporal resolution together with high sensitivity and specificity, render these devices the best candidates for *in vivo* accurate EtOH and ACD detection.

Several AEBs for *in vivo* EtOH determination are already commercially available; however since *in vivo* biosensors use is not deprived of drawbacks, the fundamentals of this technology are to be well-understood in order to obtain reproducible results (Vigneshvar et al., 2015; Weltin et al., 2016). In particular, the interactions of the implanted AEB with the biological environment may severely affect its bioanalytical performances via metabolic biofouling, electrode passivation, or biodegradation. Metabolic biofouling is probably the most important problem being able to quickly alter sensitivity, limit of detection, and linear response of the implanted device (Gifford et al., 2006; Kotanen et al., 2012). Unfortunately, biocompatibility-based issues cannot be easily circumvented and adequate pre- and post-calibration procedures are needed in order to properly evaluate *in vivo* AEBs measurements (Wilson and Gifford, 2004; Wahono et al., 2012). However it is expected that the forthcoming generation of biosensors, either based on nanoscale or polymeric materials, will greatly help reducing biocompatibility issues (Nichols et al., 2013; Weltin et al., 2014; Saxena and Das, 2016).

## Conclusion

The many attempts to quantify ACD in the brain have yielded conflicting results, mainly because of the inadequacy of the available analytical techniques. Thus, it is clear that in order to solve the puzzle of ACD as a neuroactive agent we need to use adequate analytical tools, fostering their improvement, while discarding the most problematic approaches.

Spectroscopic techniques have proved to be useful for studying *in vivo* brain EtOH kinetics, in both humans and experimental animals, but ACD measurement remains outside MRS analytical scope. Nevertheless, owing to its absolute non-invasive nature MRS provides a great opportunity for *in vivo* qualitative study of the effects of EtOH in the intact brain.

Brain microdialysis is a well-known sampling technique for *in vivo* applications. However, it is now clear that many of the features that made microdialysis so successful for *in vivo* monitoring of several neurochemicals, are of limited use when coming to *in vivo* EtOH and (especially) ACD analysis. Its invasiveness together with the low temporal resolution, and the necessity of complex analytical procedures, represent the most important problems.

Biosensors are the emerging tool for the preclinical *in vivo* study of neurochemistry. When compared to microdialysis the main advantages of AEBs are represented by their reduced invasiveness, high time resolution, and the possibility to detect analytes which cannot be studied with microdialysis. In the case of EtOH monitoring, AOX-based AEBs have proved to be capable of *in vitro* and *in vivo* detecting concentrations of EtOH in the μM range. ALDH-based ACD AEBs have not been applied for *in vivo* ACD detection yet, however *in vitro* data strongly suggest that these devices may represent the most promising opportunity for *in vivo* brain ACD detection.

## Author Contributions

PE: performed literature analysis and data collection, wrote manuscript and acted as corresponding author. MD: supervised development of work, helped in data interpretation and manuscript evaluation and editing.

## Conflict of Interest Statement

The authors declare that the research was conducted in the absence of any commercial or financial relationships that could be construed as a potential conflict of interest.
